# The Influence of the Type of Background Noise on Perceptual Learning of Speech in Noise

**DOI:** 10.3389/fnins.2021.646137

**Published:** 2021-05-03

**Authors:** Liping Zhang, Friederike Schlaghecken, James Harte, Katherine L. Roberts

**Affiliations:** ^1^Department of Otolaryngology-Head and Neck Surgery, Shandong Provincial ENT Hospital, Cheeloo College of Medicine, Shandong University, Jinan, China; ^2^Warwick Manufacturing Group, University of Warwick, Coventry, United Kingdom; ^3^Department of Psychology, University of Warwick, Coventry, United Kingdom; ^4^Interacoustics Research Unit, Technical University of Denmark, Lyngby, Denmark; ^5^Department of Psychology, Nottingham Trent University, Nottingham, United Kingdom

**Keywords:** perceptual learning, auditory training, speech in noise, generalization, babble noise

## Abstract

**Objectives:**

Auditory perceptual learning studies tend to focus on the nature of the target stimuli. However, features of the background noise can also have a significant impact on the amount of benefit that participants obtain from training. This study explores whether perceptual learning of speech in background babble noise generalizes to other, real-life environmental background noises (car and rain), and if the benefits are sustained over time.

**Design:**

Normal-hearing native English speakers were randomly assigned to a training (*n* = 12) or control group (*n* = 12). Both groups completed a pre- and post-test session in which they identified Bamford-Kowal-Bench (BKB) target words in babble, car, or rain noise. The training group completed speech-in-babble noise training on three consecutive days between the pre- and post-tests. A follow up session was conducted between 8 and 18 weeks after the post-test session (training group: *n* = 9; control group: *n* = 7).

**Results:**

Participants who received training had significantly higher post-test word identification accuracy than control participants for all three types of noise, although benefits were greatest for the babble noise condition and weaker for the car- and rain-noise conditions. Both training and control groups maintained their pre- to post-test improvement over a period of several weeks for speech in babble noise, but returned to pre-test accuracy for speech in car and rain noise.

**Conclusion:**

The findings show that training benefits can show some generalization from speech-in-babble noise to speech in other types of environmental noise. Both groups sustained their learning over a period of several weeks for speech-in-babble noise. As the control group received equal exposure to all three noise types, the sustained learning with babble noise, but not other noises, implies that a structural feature of babble noise was conducive to the sustained improvement. These findings emphasize the importance of considering the background noise as well as the target stimuli in auditory perceptual learning studies.

## Introduction

Understanding speech in noise can present a considerable challenge, even for listeners with good hearing. This challenge is exacerbated for listeners with hearing impairment, and particularly for older adults who are affected by age-related declines in both hearing and cognition (Roberts and Allen, 2016). For listeners who find it particularly difficult to understand speech amid background noise, such as those with hearing impairment or auditory processing disorder (APD) (Iliadou et al., 2017), auditory perceptual training has the potential to improve speech-in-noise comprehension (Sweetow and Palmer, 2005; Anderson et al., 2013; Weihing et al., 2015). However, auditory training does not always result in robust benefits, or generalize to untrained tasks or stimuli (Henshaw and Ferguson, 2013; Karawani et al., 2016), resulting in a need to optimize auditory training paradigms. In this study, we ask whether the type of background noise used in training affects the extent to which any improvements in speech-in-noise identification generalize to other background noises, and are sustained over time.

Improvements in auditory training paradigms could prove highly beneficial to both normal-hearing and hearing-impaired listeners. Normal-hearing listeners can experience difficulty hearing in noisy environments, and while speech recognition by people with hearing aids and cochlear implants has improved significantly over the past several years due to technological improvements, the ability of most hearing-impaired people to understand speech in noisy environments is still quite poor (Dorman and Wilson, 2004; Ricketts and Hornsby, 2005). Due to the plasticity of the auditory system (Irvine, 2018), auditory perceptual training has the potential to improve the listening performance of all listeners, and to help hearing-aid and cochlear-implant users make better use of their prosthetic device (Sweetow and Sabes, 2006; Boothroyd, 2007; Moore and Shannon, 2009). The present study investigates the effect of auditory perceptual training on normal-hearing listeners, to enable an evaluation of the effect of background noise on training outcomes in the absence of potentially confounding variables related to hearing impairment.

Auditory perceptual learning is defined as an improvement in the ability to detect, discriminate, or group sounds and speech information (Goldstone, 1998; Halliday et al., 2008). While many studies show substantially improved auditory perception following training, practical benefits to listeners will arise only if that training benefit generalizes to untrained tasks and stimuli, and is sustained over time. Generalization occurs when training on one auditory task leads to improvement on a novel auditory task (Grimault et al., 2003), and when training with one set of stimuli leads to improvements in perception of untrained, novel stimuli (Tremblay and Kraus, 2002; Davis et al., 2005; Wong and Perrachione, 2007).

The extent to which training generalizes is likely to depend on whether the untrained stimuli or task include the same internal perceptual and cognitive noise (Amitay et al., 2014) and/or decision-making processes (Jones et al., 2015; Karawani et al., 2016) as those in the training task. Many studies find that generalization of learning to untrained tasks and/or stimuli is not robust (Henshaw and Ferguson, 2013). For example, Karawani et al. (2016) found that both normal-hearing and hearing-impaired listeners benefited from speech-in-noise training, but that there was little generalization to untrained speech and non-speech sounds. An improved understanding of how and when auditory perceptual training generalizes in normal-hearing participants is needed to help devise better training programs for people with hearing impairment (Loebach et al., 2009).

Auditory perceptual learning studies frequently study identification of speech or other sounds amid background noise (e.g., babble or speech-shaped noise), because the ability to detect speech signals in a noisy environment is critical in people’s daily communication. When evaluating whether benefits of perceptual training generalize to other stimuli, these studies tend to focus on generalization to novel target speech or other sounds (Clarke and Garrett, 2004; Hervais-Adelman et al., 2011). These studies have demonstrated that there is greater generalization when training and test materials are similar (Hirata, 2004), and that high-variability training with a number of talkers leads to increased generalization to novel talkers compared with single-talker training (Clopper and Pisoni, 2004; Stacey and Summerfield, 2007; Casserly and Barney, 2017) (see Samuel and Kraljic, 2009 for a review). However, the background noise is also constantly changing in the real world, and so it is equally important that training generalizes to other types of background noise, particularly real-world environmental noise. The novelty of this study is that we aim to determine whether changing the background noise affects the amount of benefit that is maintained from auditory perceptual training.

Background noise can interfere with speech understanding through energetic masking, where the background noise has energy in the same frequency region as the speech signal, thus preventing the speech signal from being perceived. When the background noise fluctuates, as is likely with real-world environmental sounds and competing speech, the listener is afforded opportunities to “listen in the dips,” or “glimpse” the speech signal (Howard-Jones and Rosen, 1993). Alternatively, background noise can produce informational masking that results from difficulties with auditory scene analysis (Bregman, 1990), particularly when the listener has difficulty segregating target speech from the background masker (Brungart, 2001) due to failures of object formation or selection (Shinn-Cunningham, 2008).

Due to the differing effects of energetic and informational masking, the amount and type of benefit that participants receive from perceptual training may differ depending on the type of background noise. Steady-state noise, such as speech-shaped noise, is likely to provide consistent energetic masking but little informational masking. On the other hand, the temporal variation in babble-noise will afford more opportunities for glimpsing, but increased informational masking if words are partially audible. Correspondingly, training strategies that improve glimpsing or segregation may be more useful for speech presented in babble than for speech presented in steady-state noise. Van Engen (2012) trained participants on English sentence recognition in three different background noise conditions: speech-shaped noise, Mandarin babble, and English babble. She found that English sentence recognition was much better with babble background noise (both English and Mandarin) than with speech-shaped noise. The results suggest that to improve people’s speech perception in speech-in-speech environments, it is better to train them with background noise that is structured in a similar way to speech than with noise that has relatively consistent amplitude over time. Similarly, Green et al. (2019) found that training with speech-in-babble-noise improved cochlear-implant users’ perception of sentences in babble noise, but did not result in improved perception of phonemes in speech-shaped noise. These studies suggest that speech-like noise may enable listeners to develop strategies that allow them to “listen in the dips,” where energetic masking is reduced. This benefit of dip-listening appears to offset any costs associated with increased informational masking for babble noise relative to steady-state noise.

Other features of the background noise can also change the amount of perceptual learning that is obtained. Felty et al. (2009) demonstrated that listeners showed greater improvement in word recognition performance when the same sample of background babble-noise was presented on each trial, compared with when different noise samples were presented on each trial. Similar results were found in a visual texture segmentation task in which a background mask was either consistent from trial-to-trial or varied on each trial (Schubö et al., 2001). In contrast, a previous perceptual learning study with vowel-consonant-vowel (VCV) stimuli found that consonant identification improved more when stimuli were presented against a random-noise background than against a fixed-noise background (Zhang et al., 2014), indicating that the effects of background noise on perceptual learning differ with different types of target stimuli (very short VCV targets contrasting with longer word stimuli).

During everyday listening, background noise is likely to include environmental sounds (e.g., washing machine, traffic) as well as speech sounds (e.g., television, other people’s conversations). To date, though, studies that have looked at perceptual training with environmental stimuli have included environmental sounds as the target rather than background (e.g., footsteps, slamming door, air conditioner, dishwasher; see Burkholder, 2005; Reed and Delhorne, 2005; Kidd et al., 2007). A study by Loebach and Pisoni (2008) trained normal-hearing participants with a simulation of a cochlear implant. Each group was trained with one type of auditory stimulus (from a choice of words, sentences, and environmental sounds) and then later tested on all types of auditory stimuli. The results showed that all groups obtained significant improvement in the specific stimuli they were trained on. However, while perceptual learning did not transfer from training on speech to the recognition of environmental sounds, it did transfer from training on environmental sounds to both untrained environmental sounds and speech sounds. This finding suggests that there are differences in how well perceptual learning transfers from speech to environmental stimuli, and vice versa.

The present study will investigate the perceptual learning benefit obtained when participants are trained to identify Bamford-Kowal-Bench (BKB) sentences presented in background noise. Because auditory perceptual learning studies have demonstrated that word-identification training outcomes are better with word and sentence stimuli than with nonsense syllables phonemes (Stacey and Summerfield, 2008), BKB sentences were used as the target stimuli. The study has two aims: one is to investigate whether perceptual learning generalizes from the background noise used in training (babble noise) to other real-life environmental background noises (the sound of a car passing by, and rain noise), the other is to explore whether any generalization of perceptual learning is sustained over a period of several weeks. The environmental sounds were selected as examples that commonly occur in everyday life, typically persist for long enough to mask a short sentence, and do not include speech (e.g., not background television noise or music with lyrics).

## Test Methods

### Participants

Twenty-four normal-hearing native English speakers (8 males and 16 females, aged 18–33 years) participated in this study. None had previously participated in psychoacoustic experiments. Pure-tone audiometry thresholds were less than 20 dB HL at all frequencies between 250 and 8,000 Hz (BSA, 2011). Participants were not tested for auditory processing disorder (APD; Iliadou et al., 2017), but all confirmed that they were not aware of any problems with their hearing and that they had not been exposed to loud noises in the past 24 h. Participants in the training and control groups did not differ in terms of age [mean age 23.42 in the control group and 23.00 in the experimental group, *t*(22) = −0.24, *p* = 0.81]. Participants were all volunteers recruited from the student and staff population of the University of Warwick. All gave written informed consent before participating. Ethics approval for this study was given by the Biomedical and Scientific Research Ethics Committee (BSREC) of the University of Warwick.

### Test Stimuli

Bamford-Kowal-Bench (BKB; Bench et al., 1979) sentences recorded by a female British speaker were used as speech material. The speech material consisted of 21 different lists, with each list containing 16 sentences and a total of 50 target words. The sentences were centrally embedded in 2 s of background noise. The babble noise comprised 2 s of 8-speaker babble noise with four female and four male British-English speakers (Verschuur et al., 2013). Recordings of rain and a car passing by were downloaded from https://www.soundsnap.com. The signal-to-noise ratio for each sentence was determined by comparing the root mean square average amplitude of the signal file with the background noise file (the portion that actually overlapped with the sentence). The root mean square intensity was normalized to the same fixed value for all background noise.

**FIGURE 1:**
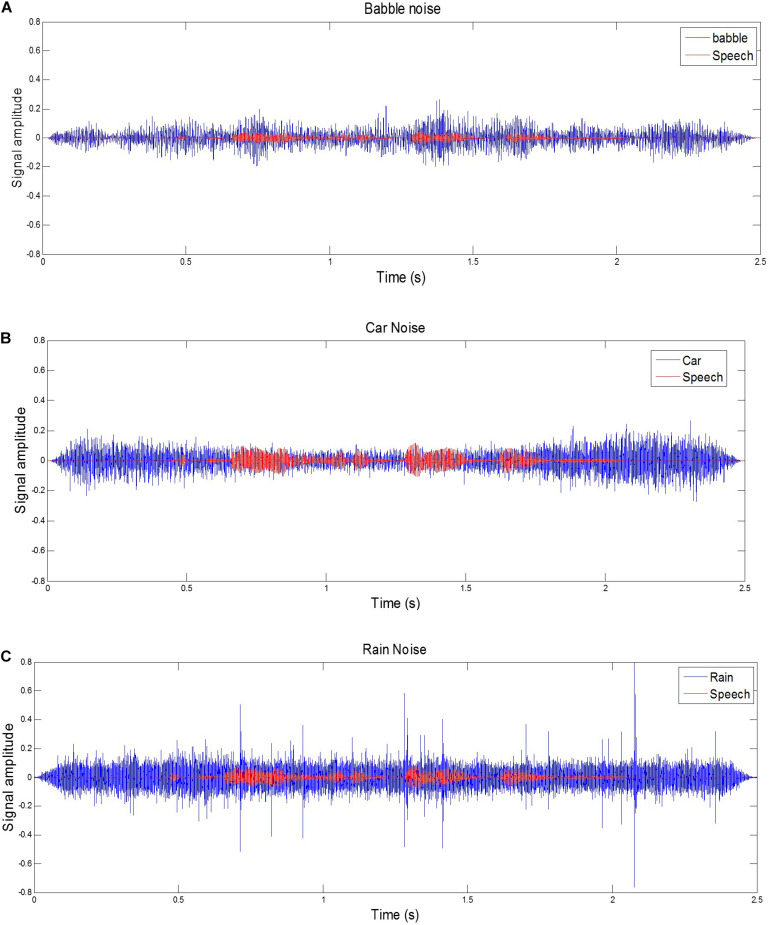
Examples of a target sentence (“The clown had a funny face”) in the background noise of babble, car and rain. The line series are shown for **(A)** target sentence in babble noise with SNR –20 dB. **(B)** target sentence in car noise with SNR –12 dB, **(C)** target sentence in rain noise with SNR –15 dB.

All sounds were presented through Sennheiser HD 580 headphones. Calibration was carried out prior to the main test. An IEC 711 acoustic coupler and a precision microphone were used to calibrate the output. Then the maximum sound pressure levels from the PC were controlled to ensure that output from the software (MATLAB) was within the exposure action value (65 dB SPL for the speech plus noise stimuli). Sampling rate for all signals was 44.1 kHz. Signal-to-noise ratios (SNRs) were fixed for a given noise type, but varied for each noise condition (babble noise −20 dB, car noise −12 dB, rain noise −15 dB). These SNR levels were based on a pilot study (*n* = 8), in which they gave approximately 50% correct target-word identification with each of the background noises. Figure 1 shows the waveforms for an example sentence “The clown had a funny face” with three kinds of background noise.

### Experiment Procedure

**FIGURE 2:**
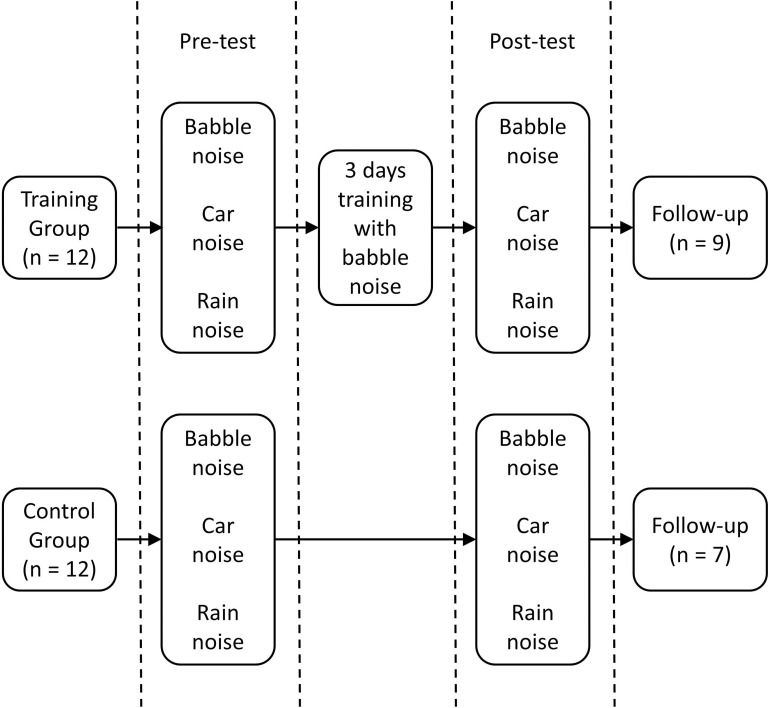
Experimental design.

All tests were carried out in a sound-attenuating room. Participants were randomly assigned to either a control (*n* = 12) or training (*n* = 12) group (see Figure 2). Before the test, a pure tone audiogram was carried out to confirm that the person qualified to participate. Participants then received written instructions and were presented with one example sentence without background noise to familiarize them with the stimuli. The participant was asked to repeat the sentence. Next, babble, car, and rain background noise samples were presented separately.

Participants were informed that during the experimental trials, the speech sounds would be softer than the background noise. Both the training and the control group completed a pre-training test session (“pre-test”) and a post-training test session (“post-test”) lasting approximately 5 min each. The training group attended three consecutive daily 30 min training sessions with BKB sentences presented amid babble noise between the pre- and post-test sessions. The control group only attended for the pre-test and post-test sessions. There was a 1 day fixed time interval between pre- and post-test session for the control group. Participants were asked to repeat each sentence and were encouraged to guess even if the sentences they repeated would result in a nonsense or incomplete sentence. All the test sessions were conducted over three consecutive days (Day 1: pre-test and training session one; Day 2: training session two; Day 3: training session three and post-test). The pre- and post-test sessions included one 16-item BKB sentence list with babble noise, one with car noise, and one with rain noise. The order of the three noise conditions was randomized in the pre- and post-tests but the BKB sentence list was the same across participants. Different BKB sentence lists were used for the pre-test (lists 1–3), training (lists 4–15), and the post-test (lists 16–18) sessions. Sessions were kept intentionally short to ensure that participant motivation remained high. The number of sessions was based on a longer study with vowel-consonant-vowel stimuli (Zhang et al., 2014) in which participants reached an asymptote after three training sessions. No feedback was given in any session.

A final follow-up test session was carried out to investigate whether training effects and generalization to other background noises could be maintained over time. Participants were recalled between 8 and 18 weeks after the post-test session [with no significant difference in the time gap between the training and control groups, *t*(14) < 1]. The procedure was the same as the pre-and post-test sessions, though a new set of BKB sentence lists were used (lists 19–21). As some of the participants had already left the university, not all the listeners attended the follow-up study. Nine training group and seven control group participants attended the follow-up test session.

## Test Results

### Pre- and Post-test Results

We calculated the number of BKB keywords that were correctly identified for each test, out of a maximum of 50. For the statistical analyses (but not graphs or reported values), the percentage correct was converted using a rationalized arcsine transform (Studebaker, 1985), using the formula for a small number of trials, to produce rationalized arcsine units (RAUs). This transformation corrects for deviations from normality while keeping the values numerically close to the originally percentages, for ease of interpretation. Where sphericity could not be assumed a Greenhouse-Geisser correction has been applied, and is evident from non-integer degrees of freedom.

**FIGURE 3:**
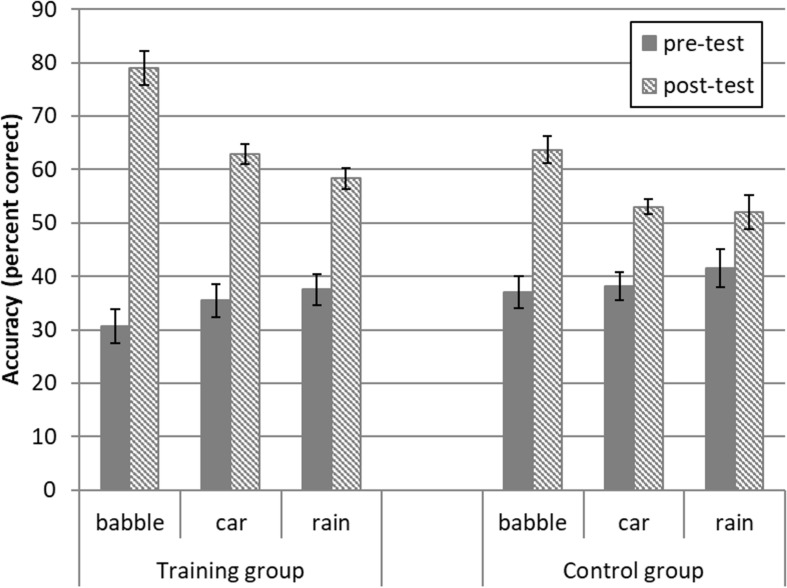
Word identification accuracy (percent correct) for the training (*n* = 12) and control (*n* = 12) groups with three different background noises. Error bars reflect ± one standard error.

Figure 3 shows word-identification accuracy in the different conditions. A 2 (group) × 3 (noise condition) analysis of covariance (ANCOVA) was used to investigate whether post-test word identification was better for the trained or control group, and whether this differed across noise conditions (babble, car, rain). Pre-test word identification was used as a covariate to control for baseline differences in pre-test performance. The ANCOVA revealed that the training group had significantly higher post-test accuracy than the control group [group, *F*_(1,19)_ = 38.56, *p* < 0.001 η^2^ = 0.67]. There was no significant difference between the three background noise conditions [noise, *F*_(1.55,29.47)_ = 2.06, *p* = 0.15, η^2^ = 0.10], but there was a significant interaction between the group and noise conditions [noise × group interaction, *F*_(1.55,29.47)_ = 5.55, *p* = 0.014, η^2^ = 0.23].

Pairwise comparisons with a Bonferroni correction for multiple comparisons (critical *p* = 0.0167) showed that the training group had significantly higher post-test scores than the control group in both the trained (babble) and untrained (car and rain) conditions, when controlling for pre-test performance (training post-test accuracy − control post-test accuracy in babble noise = 15.33, *p* < 0.001; car noise = 9.83, *p* < 0.001; rain noise = 6.33, *p* = 0.015). To probe whether training led to a greater improvement in the trained background noise than other noise types, we conducted *post hoc* (group × noise) ANCOVAs that included one pair of noise conditions at a time, still controlling for pre-test performance. A significant interaction between group and noise condition would indicate that the amount by which the training group outscored the control group was significantly greater for one type of noise than the other. The training group outscored the control group by more in the babble noise condition than the car noise [*F*_(1,20)_ = 8.11, *p* = 0.010, η^2^ = 0.29], or rain noise conditions [*F*_(1,20)_ = 6.73, *p* = 0.017, η^2^ = 0.25], but there was no difference in the pre- to post-test improvement between the car and rain noise conditions [*F*_(1,20)_ = 1.02, *p* = 0.33, η^2^ = 0.048].

For transparency, a two-way (group × noise) analysis of variance (ANOVA) was used to confirm that the improvement in RAUs from pre- to post-test was greater for the training than for the control group, and differed across the noise conditions. A main effect of group [*F*_(1,22)_ = 46.17, *p* < 0.001, η^2^ = 0.68] confirmed that from pre- to post-test, the training group showed greater improvement in word identification accuracy than the control group. There was also a significant difference between the three noise conditions [noise: *F*_(2,44)_ = 42.98, *p* < 0.001, η^2^ = 0.66], and a significant interaction between group and noise condition [group × noise: *F*_(2,44)_ = 4.21, *p* = 0.02, η^2^ = 0.16].

### Summary of Pre- to Post-test Results

The training group showed significantly better post-test word identification accuracy than the control group. This was true for both the trained (babble) noise and to a lesser extent the untrained (car and rain) background noises, showing some generalization of learning to untrained background noises. The training benefit was larger for the trained background noise, with the training group outscoring the control group by a greater amount in the babble noise condition than the car or rain noise conditions, with no difference in improvement between the car and rain noise conditions. This pattern of results was confirmed when the improvement from pre- to post-test was analyzed, with the training group showing greater improvement than the control group, with the greatest effect size for speech in babble noise.

### Individual Training Performance Across Sessions

**FIGURE 4:**
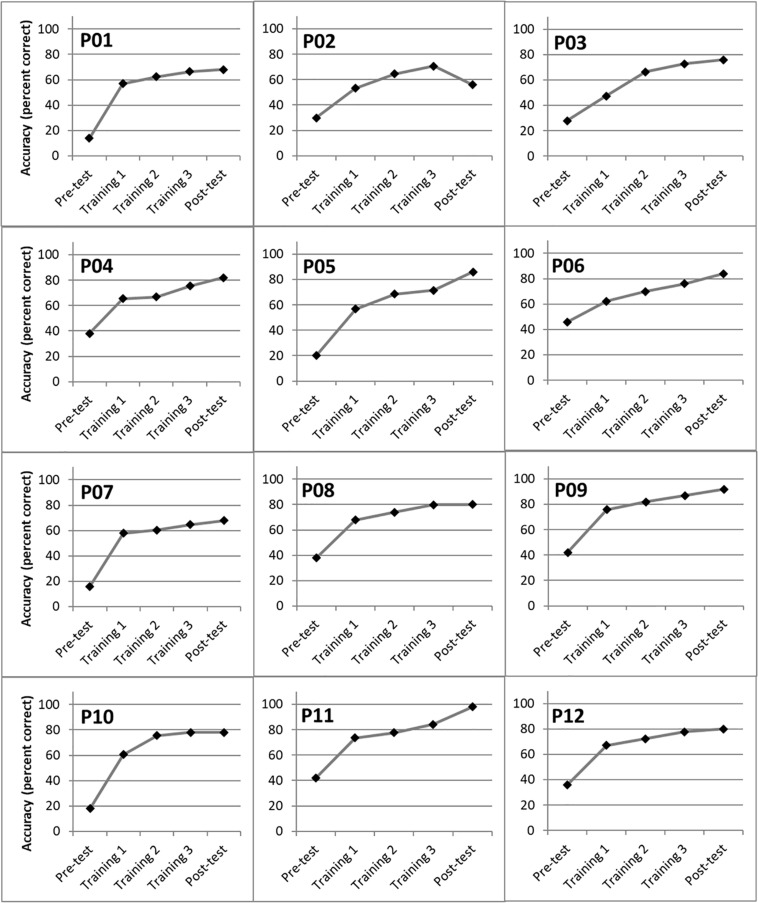
Word identification accuracy for individual members of the training group at the pre-training test (“pre-test”), Training Session 1, Training Session 2 Training Session 3, and at the post-training test (“post-test”), for the babble-noise conditions only.

Individual performance across training sessions, for the babble-noise condition only, was analyzed to confirm that the improvement in word identification had reached an asymptote. Figure 4 shows BKB word identification RAUs for each participant in the training group, in each test and training session. A one-way repeated-measures ANOVA showed that for the babble noise condition, there was an overall improvement across the pre-test, training, and post-test sessions [*F*_(2.4,26.4)_ = 127.83, *p* < 0.001, η^2^ = 0.921]. *Post hoc* t tests with a Bonferroni correction for multiple comparisons demonstrated an improvement from each session to the next (pre- to Training Session 1, Training Session 1 to Training Session 2, and Training Session 2 to Training Session 3 (*t*_11_ = 11.42, 5.39, and 10.70, respectively, all *p*s < 0.001), with the exception of the final comparison between Training Session 3 and the post-training test (*t*_11_ = 1.71, *p* = 0.12). In other words, no further improvement was found between the final training session and the post-training test.

### Results for the Follow-Up Test

**FIGURE 5:**
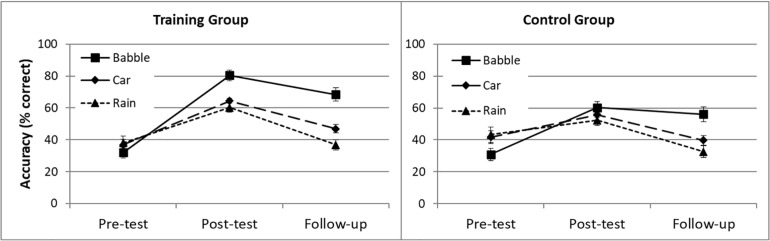
Word identification accuracy for the training (*n* = 9) and control group (*n* = 7) from three test sessions (pre-test, post-test, and follow-up test) with three different background noises (babble, car and rain noise).

Figure 5 shows word identification RAUs for the 16 participants who took part in the follow-up test. Those who participated in the follow-up session did not have significantly different pre- or post-test scores to those who did not, and did not differ in their change in accuracy from pre- to post-test in any of the background noise conditions (all ps > 0.1). A mixed ANOVA was used to analyze the word identification accuracy for both groups (training and control), in the different noise conditions (babble, car and rain), across the three test sessions (pre-test, post-test and follow-up). Overall, performance was better for the training than for the control group, resulting in a significant main effect of group [*F*_(1,14)_ = 4.75, *p* = 0.047, η^2^ = 0.253], and better in the post-test than in the pre-test or follow-up session, reflected in a significant main effect of time [*F*_(2,28)_ = 127.31, *p* < 0.001, η^2^ = 0.901]. Follow-up pairwise comparisons showed that performance improved from pre-test to post-test, then declined from post-test to follow-up, but remained better than at pre-test (all *ps* < 0.001).

Word identification accuracy differed significantly across the noise conditions [*F*_(1.45,30.30)_ = 8.24, *p* < 0.01, η^2^ = 0.371], and follow-up pairwise comparisons demonstrated that word identification accuracy was significantly better in the babble noise condition than in the car or rain noise conditions (both *ps* < 0.05), whereas the latter two did not differ substantially from each other (*p* = 0.056). There was also a significant interaction between time and noise condition [*F*_(3.40,47.57)_ = 29.57, *p* < 0.001, η^2^ = 0.679].

*Post hoc* t tests using Bonferroni correction for multiple comparisons (*p* = 0.05/9, i.e., 0.0056) showed that post-test scores were significantly higher than pre-test scores for all types of background noise (all *ps* < 0.001). For the babble-noise condition, test scores at follow-up remained significantly higher than at pre-test (*p* < 0.001), and were not significantly different from post-test (*p* > 0.0056), suggesting that the perceptual learning benefit was maintained over time. In contrast, for the car and rain noise conditions, there was a significant decrease in word identification accuracy from post-test to follow-up (both *ps* < 0.001), and scores at follow-up were not significantly different from those at pre-test (*p* > 0.0056). The interaction between test time and group was significant [*F*_(2,28)_ = 13.39, *p* < 0.001, η^2^ = 0.489], but there was no significant interaction between test time, group, and noise condition [*F*_(3.40,47.57)_ = 0.66, *p* = 0.621, η^2^ = 0.045].

### Summary of Results for the Follow-Up Test

The training group showed significantly better word identification than the control group. Overall, word identification accuracy was better at post-test than at follow-up, and better at follow-up than at pre-test, but this differed across noise conditions. In the babble noise condition, participants maintained their improved performance from post-test to follow-up, but in the car and rain noise conditions performance returned to pre-test levels. The effects of time and noise condition did not interact with group, indicating that the same pattern of results was found for both the training and control groups.

## Discussion

The present findings demonstrate that training participants to identify BKB keywords amid babble noise can improve BKB word-identification accuracy in a range of background noises. Participants who were trained to identify BKB sentences amid background babble noise had higher post-test accuracy than the control group, not only for BKB sentences presented amid babble noise, but also, to a lesser extent, for BKB sentences presented amid car and rain noise. In other words, the benefits of training participants to understand sentences amid babble noise showed some generalization to sentences presented amid car and rain noise. Nonetheless, participants showed greater improvement in the babble-noise condition than in the car- or rain-noise conditions, as would be expected given that the training sessions involved identical stimuli to the post-test babble noise condition (Morris et al., 1977; Roediger et al., 1989; Borrie et al., 2012).

Perceptual training can help listeners to make better lexical judgments about stimuli, process sound information to a higher cognitive order, and reduce participants’ attention to lower-order acoustic features (Loebach and Pisoni, 2008). All of these skills should generalize to other forms of background noise, resulting in the generalization of learning from babble noise to car and rain noise seen in the present study. In addition, earlier perceptual speech training studies with synthetic speech in quiet suggest that auditory perceptual training may adjust the auditory system by increasing awareness of informative phonetic cues, decreasing the influence of less useful stimuli, or both (Schwab et al., 1985; Francis et al., 2007; Francis and Nusbaum, 2009).

A subset of participants was followed up to determine if training benefits persisted over time. Improvements from pre- to post-test were sustained over a period of several weeks for words presented in babble noise, but not for words presented in car or rain noise, for which performance returned to pre-test levels. The sustained improvement for speech identification amid babble noise was present for both training and control groups, suggesting that the sustained improvement may relate more to the nature of babble noise than to the benefits of exposure or training *per se*. The follow-up study was based on only a subset of participants and therefore has lower power than the main analyses. *Post hoc* power analysis assessed the power for the main finding of a greater increase in word recognition from pre- to post-test for the training group compared to the control group, in babble noise. This indicated that the main study had power > 0.99 (one-tailed hypothesis; alpha = 0.05, *n* = 12 per group), whereas the same analysis for pre-test to follow-up had power of only 0.72 (*n* = 9 in the training group and 7 in the control group). Nonetheless, the finding that improvements in speech-in-noise understanding were sustained over time for speech in babble noise, but not other types of noise, raises important questions for future research into the role of the background noise.

Why might the improvement from pre- to post-test be sustained over several weeks for words presented amid babble noise but not for words presented amid car and rain noise? Participants in the control group had identical exposure to the different background noises and yet had better word identification accuracy for words in babble noise than car or rain noise at follow-up, several weeks after the initial study. Neuroimaging studies demonstrate that speech and environmental stimuli show overlapping patterns of activation (Lewis et al., 2004; Loebach and Pisoni, 2008), and share the same auditory sound processing pathway leading to sound recognition (Kidd et al., 2007). However, it remains a controversial discussion regarding whether specific regions of the auditory cortex are selectively involved in processing speech. Overath et al. (2015) have argued there are structures in the auditory brain tuned for speech-specific spectro-temporal structure.

One simple possibility for the different levels of sustained learning is that babble noise affords more opportunities than steady-state noise for “glimpsing” (listening in the comodulated or uncomodulated dips; Rosen et al., 2013). While dips are present in the car and rain noise samples, they are less frequent and with reduced amplitude modulation (see Figure 1). Potentially, through exposure and/or training, participants learned to utilize dips more effectively, and this specific learning was sustained over time, benefiting the babble noise condition but not the car and rain noise conditions.

Understanding speech presented amid other speech sounds relies on being able to segregate the target stream from the background speech sounds (Brungart et al., 2001). Training with speech in babble noise can help listeners to “pick up” target sounds and “tune out” particular sorts of background noise (Van Engen, 2012). Listeners in the current study may make use of the speech cues (speech spectral components) in babble noise to pick up the target speech information and tune out the irrelevant sound information. Previous work has shown that auditory perceptual training can affect the distribution of attention to speech stimuli by training participants to inhibit the irrelevant sound cues, resulting in reduced processing of the unattended stimuli (Melara et al., 2002; Loebach and Pisoni, 2008; Tremblay et al., 2009; Murphy et al., 2017).

There are of course many differences in the structure over both time and frequency of the three background noises tested in this study. An active area of research in recent years (e.g., McDermott and Simoncelli, 2011) focuses on the way the brain processes and characterizes sound from the environment (e.g., rain, ocean waves, waterfall, traffic, swarms of insects, etc.). In the context of the present study we would refer to these as background noises. Sound textures are believed to be represented in the brain by statistics that summarize signal properties over space and time (McWalter and McDermott, 2018). It has been shown that when processing the underlying statistical structure of sounds, different strategies are used by the auditory system. For example, the time over which the brain integrates information on these sound statistics seems to adapt or vary for different sound textures (McWalter and McDermott, 2018). How training to ignore or supress one sound texture might generalize to another is, to the authors’ knowledge, unknown and largely unexplored. A potentially interesting line of research might be to determine what degree of similarity between noise/sound textures is needed to observe generalization. How one defines similarity (i.e., decides what statistical dimension to explore) for such a study is however not trivial.

Regarding the specific effects of training, it is interesting to note that participants in the training group improved in BKB word identification accuracy from pre-test to the first training session, and in each subsequent training session, but that there was no additional benefit from the third training session to the post-test. Many participants showed a sharp rise in word identification accuracy from pre-test to Training Session 1 (Figure 4), which is likely to result from increased familiarity with the test procedure and stimuli. Further gains during the training sessions are likely to result from participants learning to identify the speech sounds amid the background noise. Participants appear to have reached asymptotic performance following the three training sessions as no further benefits were found between Training Session 3 and the post-training test.

The present study found training benefits in normal-hearing young adults over a small number of short sessions. While these short sessions were sufficient to demonstrate an impact of the type of background noise on perceptual learning, longer training interventions may be needed to induce plasticity-related improvements in hearing-impaired individuals, such as hearing-aid and cochlear-implant users, or people with Auditory Processing Disorder (APD). For example, auditory training programs designed to alleviate auditory processing difficulties in APD typically involve multiple sessions per week (Weihing et al., 2015).

Participants in the control group did not attend or complete an alternative task between the pre- and post-test session. Some of the post-test benefit for the training group may therefore be due to factors unrelated to perceptual learning, such as increased concentration or familiarity with the content of BKB sentences or test environment. In future studies, an active control group could be included to identify the contribution of these factors to perceptual learning with different background noises, and to ensure equal exposure to BKB sentences across training and control groups. It is noteworthy that even without an active task, the control group showed prolonged benefits from simple exposure to speech in babble noise, but not other types of noise. One other key difference between the training and control groups is the gap prior to the post-training test session. Control participants returned after 1 day to complete the “post-training” session, whereas training-group participants completed the post-test session immediately after the Day 3 training, which potentially impacted their concentration or memory of the task. However, we are confident that these timing differences did not influence the overall results because there was no significant change for the training group between Day 3 training (1 day after the previous session) and the post-training test.

Participants gained a greater learning benefit for speech in babble noise than for speech in car or rain noise. While this is likely to be due to the increased practice with the specific speech-in-babble noise stimuli, an alternative is that the improved speech perception in the babble noise condition reflected increased familiarity with the target stimuli (Brungart et al., 2001). This increased familiarity may have proved particularly beneficial in the babble noise condition due to the increased perceptual similarity between the target speech and background noise. One way to evaluate this possibility would be to train participants with speech in either car or rain noise and determine whether benefits are still enhanced for the speech-in-babble noise stimuli.

The present study provides evidence that the background noise should be considered in the development of speech-in-noise training paradigms, and could prove valuable in providing auditory training to participants with normal pure-tone audiometry thresholds. However, further research is needed to investigate how the background noise affects perceptual learning in participants with hearing difficulties, including hearing-aid and cochlear-implant users and those with auditory processing disorder (APD). People with APD can have particular difficulty understanding speech in noise, despite normal pure-tone thresholds in quiet (Iliadou et al., 2017), and so it may prove particularly important to understand the role of the background noise when developing auditory training paradigms for this group.

This study showed that benefits of training in babble noise generalized to car and rain background noise, in the short term at least. However, all three types of noise were broadly similar in their spectral and temporal profile. Future research could investigate whether learning benefits generalize to familiar noises with different spectro-temporal profiles (e.g., drumming), or whether generalization depends on perceptual similarity.

## Conclusion

The present study has demonstrated that training participants to understand speech in babble noise leads to improved word identification accuracy. The benefits of training generalized, to some extent, to speech presented in car noise and rain noise, but were greatest for speech presented in babble noise. For both the training and control groups, improvements from the pre-training test to the post-training test were maintained over time for words presented in babble noise, but not for words presented in car and rain noise. As the sustained learning with babble noise, but not other noises, was observed in both groups, this implies that a structural feature of the babble noise (i.e., temporal fluctuations in envelope, residual pitch cues or some other feature) was conducive to sustained improvement in the BKB sentence task. This must be the case as the control group received equal exposure to the three noise types.

This study highlights the need to consider both the target and background sounds when creating auditory training programs. The outcomes provide important evidence for the use of background noise in perceptual auditory training programs to improve people’s listening ability in challenging environments. In the future, it will be worth putting together the findings from this study with other auditory perceptual learning research (Kim et al., 2006; Wong et al., 2010; Parbery-Clark et al., 2011) to explore more methods to improve speech understanding in people who experience auditory perceptual difficulties. These findings could be used as a baseline for further training for related auditory plasticity research in hearing impaired people, such as effects of age and hearing loss level on speech perception in noise (Dubno et al., 1984; Helfer and Wilber, 1990; Anderson et al., 2013; Henshaw and Ferguson, 2013; Alain et al., 2014; Karawani et al., 2016), or speech understanding in children with learning difficulties such as Auditory Processing Disorder (APD) (Bradlow et al., 2003; Ziegler et al., 2005, 2009).

## Data Availability Statement

The raw data supporting the conclusions of this article will be made available by the authors, without undue reservation.

## Ethics Statement

The studies involving human participants were reviewed and approved by the Biomedical and Scientific Research Ethics Committee (BSREC) of the University of Warwick. The participants provided their written informed consent to participate in this study.

## Author Contributions

LZ set up the study, tested participants, and analyzed the data. All authors discussed the findings, contributed to writing the manuscript, helped to design the study, and approved the final version.

## Conflict of Interest

The authors declare that the research was conducted in the absence of any commercial or financial relationships that could be construed as a potential conflict of interest.
